# Characterization of MicroRNA and Gene Expression Profiles Following Ricin Intoxication

**DOI:** 10.3390/toxins11050250

**Published:** 2019-05-02

**Authors:** Nir Pillar, Danielle Haguel, Meitar Grad, Guy Shapira, Liron Yoffe, Noam Shomron

**Affiliations:** Sackler Faculty of Medicine, Tel Aviv University, Tel Aviv 69978, Israel; nirpillar@gmail.com (N.P.); Haguel@mail.tau.ac.il (D.H.); meitargrad@gmail.com (M.G.); guyspersonal@gmail.com (G.S.); lironyoffe@gmail.com (L.Y.)

**Keywords:** ricin, microRNA, lung intoxication

## Abstract

Ricin, derived from the castor bean plant, is a highly potent toxin, classified as a potential bioterror agent. Current methods for early detection of ricin poisoning are limited in selectivity. MicroRNAs (miRNAs), which are naturally occurring, negative gene expression regulators, are known for their tissue specific pattern of expression and their stability in tissues and blood. While various approaches for ricin detection have been investigated, miRNAs remain underexplored. We evaluated the effect of pulmonary exposure to ricin on miRNA expression profiles in mouse lungs and peripheral blood mononuclear cells (PBMCs). Significant changes in lung tissue miRNA expression levels were detected following ricin intoxication, specifically regarding miRNAs known to be involved in innate immunity pathways. Transcriptome analysis of the same lung tissues revealed activation of several immune regulation pathways and immune cell recruitment. Our work contributes to the understanding of the role of miRNAs and gene expression in ricin intoxication.

## 1. Introduction

Ricin toxin, derived from the castor bean plant Ricinus communis, is a highly toxic protein that belongs to the type 2 ribosome-inactivating protein (RIP) family [1,2]. The catalytically active subunit of ricin translocates into the cell cytoplasm where it causes irreversible inhibition of protein synthesis and ultimately cell death [3]. Several methods for developing simple, reliable, and sensitive detection approaches towards ricin have been studied [4] While many of the methods for ricin detection are robust, each has limitations in selectivity, and no single approach is currently able to fully distinguish ricin from other harmful toxins [4].

MicroRNAs (miRNAs) are small, endogenous RNA molecules, 21–24 nucleotides (nt) long. They play an important regulatory role by inducing posttranscriptional gene silencing of their mRNA targets, and thereby function as negative gene expression regulators. miRNAs contribute to the regulation of most biological processes and also influence numerous pathological states, including atherosclerosis [5], kidney diseases [6], cancer, and infectious disease [7]. Several features of miRNAs render them ideal biomarkers for toxicology. These include improved stability in biofluid samples [8], tissue specific patterns of expression [9], and highly-developed multiplexing measurement methods [10]. Although various approaches towards developing simple, reliable, and sensitive methods for ricin detection have been investigated [4,10], miRNAs have been underexplored. In this study, we aimed to examine changes in the miRNA expression profile in mice following pulmonary ricin intoxication, and the application of these changes to ricin detection. We assumed that differentially expressed miRNAs will enhance our understanding of the ricin toxicity mechanism and may be used for intoxication. We detected significant changes in miRNA expression levels following ricin exposure, including distinct enrichment of innate immunity related miRNAs. Gene expression analysis further corroborated these results, and revealed the activation of several immune regulation pathways and immune cell recruitment after ricin exposure. We believe the changes revealed following ricin exposure will set the ground for a better understanding of the intoxication process.

## 2. Results

**Figure 1:**
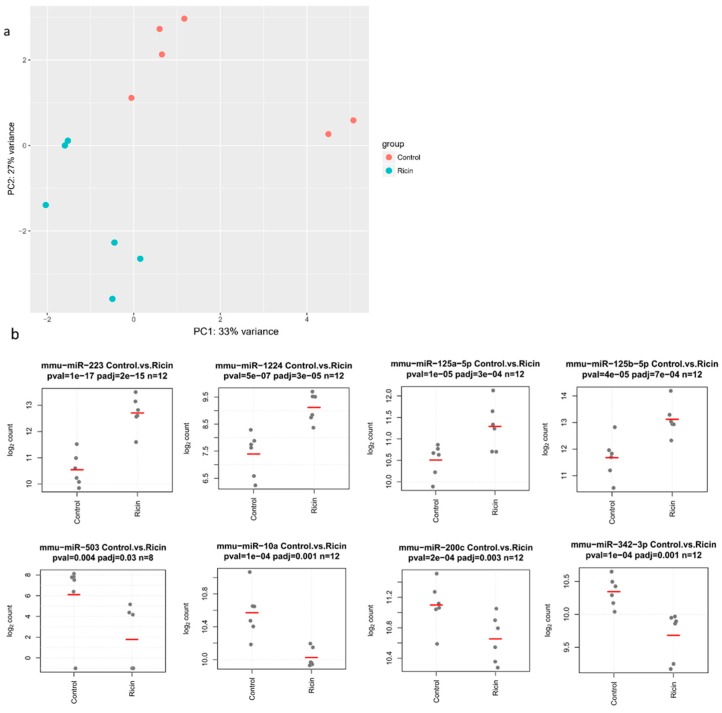
miRNA expression, ricin versus control treated. (**a**) PCA analysis of miRNA expression. The ricin and control groups are clearly distinguished by their miRNA expression profiles, as demonstrated by unsupervised clustering. (**b**) Expression of the top eight differentially expressed (DE) miRNAs. Each gray dot represents one sample. The red line indicates mean expression. All miRNAs had adjusted *p*-values <0.05.

To assess the effect of ricin inhalation on miRNA expression, 12 mice were intranasally challenged with a lethal dose of ricin or saline as a negative control. Twenty-four h after exposure, lungs were excised and total RNA was purified for multiplex miRNA profiling using the Nanostring nCounter system. Of the 600 mature mouse miRNAs probed, 182 had expression levels above the negative control probes (Table S1a). A total of 21 miRNAs were found to be deregulated in the ricin group compared to control samples (Table S2), 9 were significantly downregulated, and 12 were significantly upregulated, all with *p*-values of <0.05. Principal component analysis (PCA) using the differentially expressed (DE) miRNAs revealed sample clustering according to ricin exposure status (Figure 1). We used qPCR to validate the Nanostring results (Figure S1). To further corroborate our findings, the ricin exposure experiment and lung RNA extraction were repeated with an additional set of mice (*n* = 12). Five miRNAs (miR-223, miR-1224, miR-503, miR-10a, and miR-200c) exhibited statistically significant changes in expression that matched the results of the first Nanostring analysis.

**Figure 2:**
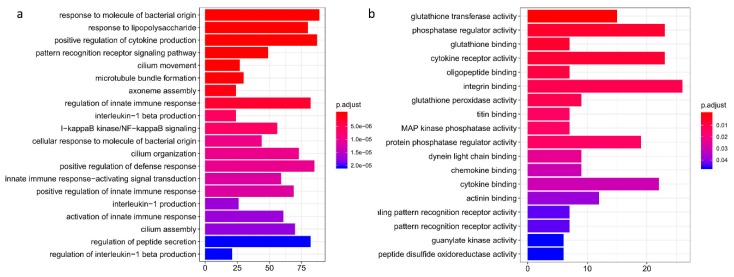
Gene ontology enrichment analysis of ricin intoxication. Barplot representation of the gene ontology biological function (**a**) and molecular processes (**b**) analysis of ricin induced transcriptome. The analysis comprised 2236 genes that had over 1-fold change expression between the ricin and control groups, with adjusted *p*-value <0.05. Both biological and molecular analyses showed enrichment for immune cell recruitment and cellular response to infection. The X-axis describes the number of genes involved in each process.

Since ricin has been shown to induce rapid, massive migration of inflammatory cells—predominantly innate immune cells [11]—we utilized the innate DB database [12], which aims to capture improved coverage of the innate immunity interactome. A total of 54 immune miRNAs appear in the database. Of these, three (miR-223, miR-10a, miR-200c) were differentially expressed in our cohort. This number was significantly higher than expected by chance (*p*-value <0.005). To strengthen the immune regulation enrichment observation and to delineate its deregulated pathways, total RNA (the same RNA used for the first miRNA multiplex analysis) was sent for RNA-sequencing (RNA-seq). Major differences in gene expression were detected between the groups, with significant expression downregulation (*p* < 0.05) of 2823 genes and upregulation of 3147 genes (Table S1b). The ricin and control groups were distinctly discriminated by their gene expression (Figure S2). These vast differences in gene expression concur with previous descriptions of transcriptome analyses of models of acute lung injury mice [13]. For ontology analysis of genes that presented with statistically significant differences, high variation in expression (absolute fold-change ≥1) revealed enrichment for immune cell responses after exposure to pathogenic agents, including IL-1 and NF-kB pathway activation, leukocyte chemotaxis and migration, and chemokine activation (Figure 2). Utilizing validated miRNA–target interactions databases, we noted that many of the differentially expressed RNA-seq genes were directly regulated by the deregulated five miRNAs (Table 1).

**Figure 3:**
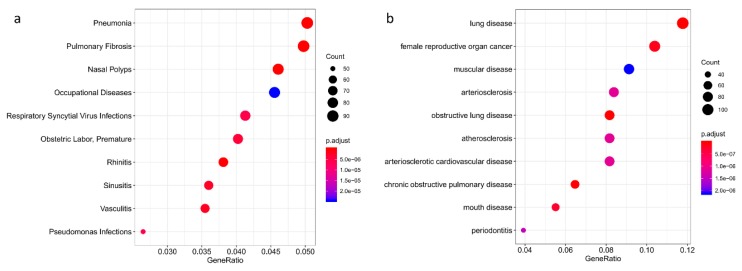
Disease enrichment analysis of ricin intoxication. Dotplot representation of ricin intoxication disease ontology. The analysis comprised 2,236 genes that had over 1-fold change expression between the ricin and control groups, with adjusted *p*-value < 0.05; these genes were evaluated for over-presentation in (**a**) PubMed and MEDLINE databases and (**b**) Disease Ontology. GeneRatio is the number of genes involved in the specific process divided by the total number of genes (2,236). Dot sizes (“count”) represent the total number of predicted gene targets of the total number of genes that are known to be involved in the listed processes and dot color indicates statistical significance. Enrichment of lung related diseases of infectious and immune natures can be detected.

**Figure 4:**
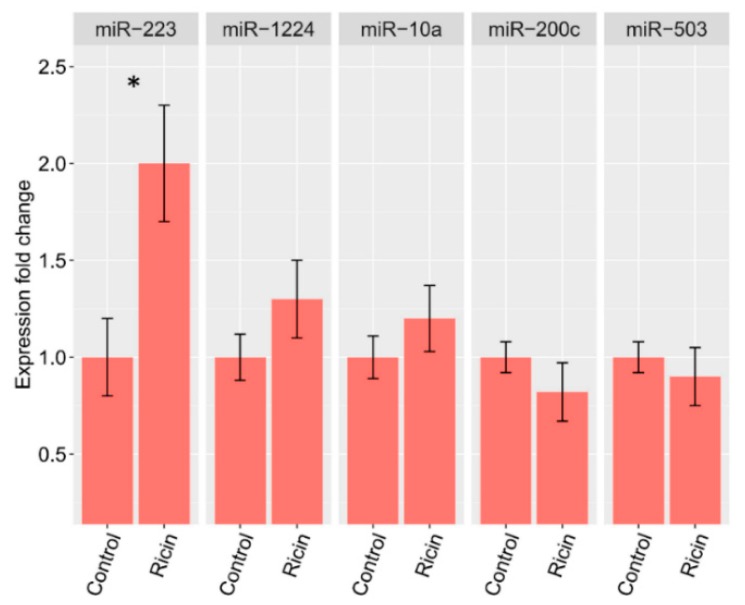
Relative expressions of miR-223, miR-1224, miR-10a, miR-200c, and miR-503 in peripheral blood mononuclear cells (PBMCs). Expression levels of miR-223, miR-1224, miR-10a, miR-200c, and miR-503 in PBMCs of ricin and control groups. miR-223 was found to be significantly upregulated in the ricin group, similar to its expression pattern in lung tissue. miR-1224, miR-10a, miR-200c, and miR-503 expression was not significantly different among the study groups. Data are represented as mean ± SEM. * *p* < 0.05.

Next, we identified the diseases with over-presentation of the same highly changed genes, using disease ontology and MEDLINE/PubMed indexed articles. Enrichment in lung-related diseases of both infectious and immune related origin was detected (Figure 3). With the aim of translating these results to a clinical setting, we examined whether the ricin induced lung miRNA changes could also be detected in peripheral blood mononuclear cells (PBMCs) as a surrogate tissue that can be safely obtained from patients. PBMCs provide a large pool of gene transcripts that have demonstrated the potential to be highly sensitive to the disease microenvironments on a system-wide basis [15,16]. PBMCs were isolated from mouse blood after intranasal ricin or saline exposure. RNA was extracted and expression levels of miR-223, miR-1224, miR-10a, miR-200c, and miR-503 were evaluated using real time PCR. miR-223 was significantly upregulated in the ricin group, similar to its trend of expression in the lungs. However, other miRNAs did not present the same expression pattern (Figure 4).

## 3. Discussion

In the present study, we explored changes in miRNA expression profiles following pulmonary ricin intoxication. We detected 21 DE miRNAs (9 upregulated and 12 downregulated) in mouse lung tissues 24 h after ricin exposure. Five miRNAs (miR-223, miR-1224, miR-503, miR-10a, and miR-200c) had similar changes in expression in a validation cohort. Next, we utilized the InnateDB dataset, which has been developed to facilitate system level investigations of the mammalian (human, mouse, and bovine) innate immune response [12], and noted significant enrichment of the DE miRNA in the InnateDB dataset (*p*-value <0.005). This stands in agreement with previous studies that explored ricin-induced lung injury and innate immune response [11,20]. Associations of miR-223, miR-1224, miR-10a, miR-200c, and miR-503 with immune regulation were previously established. miR-223 was implicated in polymorphonuclear cell development and function [21]. Further, miR-223 may play a crucial role during granulopoiesis [22]; it is upregulated throughout granulocyte differentiation and is the first miRNA that was found to dramatically alter granulocyte fate [23]. miR-223 critically fine-tunes myeloid cell activity and is involved in inflammatory diseases by regulating multiple gene transcripts including E2F1, NOD-like receptor activation, and the NF-κB pathway [24]. Overexpression of miR-223 was demonstrated to dampen acute lung injury [25]. miR-1224 was previously shown to regulate tumor necrosis factor-α (TNF-α) gene activity and miR-1224 expression was demonstrated to inversely affect LPS-induced TNF-α mRNA expression [26]. Macrophages infected with mycobacterium demonstrated significantly higher miR-1224 expression, suggesting a potential role of miR-1224 in host responses upon mycobacterium infection [27]. miR-10a negatively regulates Bcl-6 expression in T cells and has an inverse effect on germinal center reactions [28]. miR-10a is involved in stabilizing the expression of Foxp3 in regulatory T cells [29] and can suppress proinflammatory monocytic cell activation by inhibiting the activation of the proinflammatory nuclear factor κB pathway [30]. miR-200c directly regulates expression of IL-6, IL-8, and CCL-5 transcripts by binding to their 3’UTRs [31]. It suppresses signaling pathways leading to NF-κB activation after TLR4 ligation; miR-200c mimics have been shown to cause decreased expression of transcripts encoding MyD88 and to induce the expression of inflammatory molecules in response to LPS [32]. Reduced miR-503 expression augments lung fibroblast VEGF production and promotes lung fibrosis [33]. Downregulation of miR-503 in bone marrow-derived mesenchymal stem cells was linked to attenuation of lung injury after infection [34], and miR-503 levels were found to be reduced in acute lung injury [35].

Lee et al. explored miRNA expression in acute lung injury (induced by LPS administration) and demonstrated significant changes in miR-223 and miR-503 expression, as well as in six other miRNAs not shared with our expression profile [17]. These discrepancies may be related to the vast differences between the ricin and LPS immune stimulation mechanisms [36]. Vaporidi et al. discovered miRNA expression profiles in ventilator-induced lung injury [18], which included changes in 65 miRNAs, among them miR-223, miR-503, and miR-200c. This abundance of deregulated miRNAs compared to our results could be explained by the pathologic differences in mechanic and pathogenic etiology of acute lung injury [37] and supports the notion that changes in miRNA expression in acute lung injury differ by their causative agents (also see Table 2).

To functionally validate the results of the miRNA expression analysis and to decipher its biological relevance, we investigated the lung transcriptome of mice exposed to ricin. Enrichment of immune regulation mediators and pathways, such as cellular response to bacterial stimulus, IL-1 and NF-kB signaling, leukocyte migration, and chemokine activity were discovered in biologic and molecular gene ontology analyses. Lung infections and immune related pathologies, such as chronic obstructive pulmonary disease, asthma, and bacterial infections were seen when utilizing the disease ontology database and PubMed indexed articles. Taken together, the miRNA expression and its transcriptome counterpart highlight the alteration in immune response following ricin intoxication. 

PBMCs are a promising option for assessment of biologically distinct responses to various pathologies when the affected organs cannot be biopsied without further compromising the patient’s health. Specifically, genomic analysis of PBMCs has been shown to distinguish between several lung related pathologies, including lung cancer [38], asthma [39], and pneumonia [40]. Here, we observed little resemblance between PBMC miRNA profiles and lung miRNA expression, with only the miR-223 PBMC profile mimicking its lung profile. A possible explanation for this is that most of the miRNAs expressed in ricin-induced lung toxicity originate in lung parenchyma (mostly lung epithelial cells) and the supporting stromal cells (fibroblasts, dust cells) and hence cannot be fully detected in PBMCs. Another potential explanation is that the time points used in this study are not conducive to a correlative observation between lung and PBMC miRNA responses. Future studies would benefit from a time-course assessment of miRNA profiles in response to ricin-induced intoxication.

A major limitation of our study is the usage of a rodent model to study human toxicity. Due to the complexity of running these experiments in humans, we cannot extrapolate our results to such a context. Nonetheless, we feel that our work represents an important attempt to understand the miRNAs and mRNAs modified during ricin intoxication in animals. Given that all five identified miRNAs are conserved in mammals, it is plausible that the outcome may have similarities among organisms of this class. Major strengths of our study include the comprehensive evaluation, identification, and validation of all miRNA and mRNA expression levels, and also the network and regulation analysis.

In conclusion, we describe a unique lung miRNA expression profile of pulmonary ricin intoxication that was validated in two separate in vivo experiments. The DE miRNAs detected are enriched for innate immune response and elucidate the regulatory roles of miRNAs following ricin exposure, as was further supported by transcriptome analysis. Additional studies should be conducted to further characterize the miRNA regulatory networks and to translate these findings into a miRNA-based diagnosis.

## 4. Materials and Methods

### 4.1. Animal Studies and Ricin Intoxication

Ricin preparation and animal experiments were performed at the Israel Institute for Biological Research as described previously [41]. All animal experiments were performed in accordance with Israeli regulations and were approved by the Ethics Committee for Animal Experiments at the Israel Institute for Biological Research (protocol identification code M-18-2016, date of approval: 8 September 2016). Treatment of animals was in accordance with regulations outlined in the USDA Animal Welfare Act and the conditions specified in the Guide for Care and Use of Laboratory Animals (National Institute of Health, 1996). Lung and blood samples were collected from ricin-intoxicated mice at 24 h after intranasal exposure to 7 μg/kg of crude ricin. Whole blood (0.5 mL/mouse) was extracted and treated with RBC lysis buffer (Sigma-Aldrich, St. Louis, MO, USA) followed by centrifugation of 1200 × g for 20 min to isolate PBMCs. Lungs and PBMCs were stored at −80 °C.

### 4.2. RNA Extraction

Lung tissues were homogenized using the TissueLyser LT (QIAGEN, Hilden, Germany), and total RNA from homogenized lung tissues and PBMCs were extracted using TRIzol reagent according to the manufacturer’s instructions (Invitrogen, Thermo Fisher Scientific, Waltham, MA, USA). The final RNA concentration and purity were measured using a NanoDrop ND-1000 spectrophotometer (NanoDrop Technologies, Thermo Fisher Scientific).

### 4.3. Nanostring miRNA Expression Assay

The multiplexed NanoString nCounter miRNA expression assay (NanoString Technologies, Seattle, WA, USA) was used to profile 600 mouse miRNAs. The assay was performed according to the manufacturer’s protocol. Briefly, at least 100 ng of total RNA was used as input material, with 3 μL of the threefold-diluted sample. A specific DNA tag was ligated to the 3’ end of each mature miRNA, providing unique identification for each miRNA species in the sample. The tagging was performed in a multiplexed ligation reaction utilizing reverse complementary bridge oligos to achieve ligation of each miRNA to its designated tag. All hybridization reactions were incubated at 64 °C for 18 h. Excess tags were then washed, and the resulting material was hybridized with a panel of fluorescently labeled, bar-coded reporter probes specific to the miRNA of interest. Abundances of miRNAs were quantified on the nCounter Prep Station by counting individual fluorescent barcodes and quantifying target miRNA molecules present in each sample.

### 4.4. Real Time PCR

The cDNA for miRNA was synthesized from total RNA. Reverse transcription of specific mature miRNAs was performed using TaqMan miRNA assays according to the manufacturer’s protocol (ABI). The PCR amplification and reading were conducted in triplicate using the StepOnePlus thermal cycler (ABI). Mature miRNA expression was quantified under the following thermal cycler conditions: 2 min at 50 °C, 10 min at 95 °C and 40 amplification cycles (15 s at 95 °C and 1 min at 60 °C). Expression values were calculated based on the comparative threshold cycle (Ct) method. U6 snRNA was used for miRNA levels normalization.

### 4.5. RNA-Seq Preperation

RNA-seq reads were mapped to the *Mus Musculus* reference genome GRCm38 using STAR v2.4.2a [42], supplied with gene annotations downloaded from Ensembl release 82. Expression levels for each gene were quantified using HTseq-count [43]. Samples were classified as ricin or control, with 3 replicates per group. Differential expression analysis was performed using DESeq2 [44] *R* package.

### 4.6. Data Analysis

All statistical analyses were done using R software version 3.3. NanoString data preprocessing and normalization, followed by differential expression analysis, were performed using the DEseq2 [44] package and in-house scripts [45]. The mean value of negative controls was set as the lower threshold for each sample; only miRNAs with at least 50% of their values above the lower threshold were included in downstream analysis. miRNAs displaying absolute fold-change ≥1 with a false-discovery-rate [FDR] of 5% were considered to be differentially expressed. Gene and disease enrichment analyses were performed using the clusterProfiler [46] package. miRNA–target interaction analysis was done using the multimiR package [47].

## Figures and Tables

**Table 1:** Differentially expressed miRNAs that overlapped between both in vivo experiments (12 + 12 mice) and their differentially expressed target genes. Differentially expressed genes were taken from our RNA-sequence (seq) analysis. Validated targets were imported from miRTarBase [14].

miRNAs	Adjusted *p*-value	Mean Control Expression	Mean Ricin Expression	Differentially Expressed Target Genes
mmu-miR-223	1.75 × 10^−15^	1749.90	8623.46	*Bdp1, Hpcal4, Tspyl3, Dusp8, Lif, Fmnl2, Kcnj3, Zfp467, Dbn1, Ppp4r2, F3, Pcdh17, Pvt1, Ank3, Tppp, Lonrf3, Fam13a, Ptbp2, Ankrd17, Scd1, Pdia6, Mt2, 3110043O21Rik, Rsrc2, Rest, Prkar2b, Serf1, Ntrk2, Jmjd1c, Enc1, Pitpnm3, Sgsm1, Nrep*
mmu-miR-1224	0.0000345	195.87	691.35	*Rhod, Clk4*
mmu-miR-10a	0.00136	1514.00	1318.02	*Ptpn2, Ccl9, Cenpl, Car8, Mmp25, Sgms2, Eno2, Rnd1, Tnfrsf10b, Fam227a, Tbc1d24, Gaa, Aco1, Mcc, Stam, Decr2, Rhbdl3, Ajuba, Shroom1, Ramp1, Klhl41, Ecm2*
mmu-miR-503	0.0297	190.89	23.16	*Creb5, Ncl, Inhbb*
mmu-miR-200c	0.0027	2223.93	1981.17	*Jun, Ikzf5, Map2, Sox2, Zeb2, Mgat3*

**Table 2:** Comparison of miRNAs in studies of injured lungs.

Major miRNAs Changed	Study Description	miRNAs Overlapped with Our Study	Reference
miR-223, miR-1224, miR-503, miR-10a, miR-200c	Ricin exposure to the lungs	—	Current study
miR-142, miR-98, miR-541, miR-503, miR-653, miR- 223, miR-323, miR-196b	LPS-induced acute lung injury	miR-223, miR-503	[17]
miR-155, let-7a, let-7b, miR-125b, miR-146, miR-106a, miR-543, miR-106a, miR-7, miR-135, miR-21, miR-345, miR-223, miR-24, miR-132, miR-9, miR-503, miR-211, miR-676, let-7a, miR-200c	Ventilator-induced lung injury	miR-223, miR-503, miR-200c	[18]
miR-484, miR-425, miR-96	Mycobacterium infection (in serum)	—	[19]

## Supplementary Materials

The following are available online at https://www.mdpi.com/2072-6651/11/5/250/s1, Figure S1: qPCR validation of Nanostring results. Figure S2: PCA analysis of mRNA expression. Table S1: (a) Expression comparison of all expressed miRNAs between ricin and control groups. (b) Expression comparison of all expressed mRNAs between ricin and control groups. Table S2: Differentially expressed miRNAs between ricin and control groups.

## References

[1] Olsnes S., Kozlov J.V. (2001). Ricin. Toxicon.

[2] Yermakova A., Mantis N.J. (2011). Protective immunity to ricin toxin conferred by antibodies against the toxin’s binding subunit (RTB). Vaccine.

[3] Gal Y., Mazor O., Falach R., Sapoznikov A., Kronman C., Sabo T. (2017). Treatments for Pulmonary Ricin Intoxication: Current Aspects and Future Prospects. Toxins.

[4] Bozza W.P., Tolleson W.H., Rivera Rosado L.A., Zhang B. (2015). Ricin detection: Tracking active toxin. Biotechnol. Adv..

[5] Novák J., Olejníčková V., Tkáčová N., Santulli G. (2015). Mechanistic Role of MicroRNAs in Coupling Lipid Metabolism and Atherosclerosis. Adv. Exp. Med. Biol..

[6] Metzinger-Le Meuth V., Metzinger L. (2019). miR-223 and other miRNA’s evaluation in chronic kidney disease: Innovative biomarkers and therapeutic tools. Non-coding RNA Res..

[7] Staedel C., Darfeuille F. (2013). MicroRNAs and bacterial infection. Cell Microbiol..

[8] Cortez M.A., Bueso-Ramos C., Ferdin J., Lopez-Berestein G., Sood A.K., Calin G.A. (2011). MicroRNAs in body fluids—The mix of hormones and biomarkers. Nat. Rev. Clin. Oncol..

[9] Guo Z., Maki M., Ding R., Yang Y., Zhang B., Xiong L. (2014). Genome-wide survey of tissue-specific microRNA and transcription factor regulatory networks in 12 tissues. Sci. Rep..

[10] Kolbert C.P., Feddersen R.M., Rakhsha F., Grill D.E., Simon G., Middha S., Jang J.S., Simon V., Schultz D.A., Zschunke M. (2013). Multi-Platform Analysis of MicroRNA Expression Measurements in RNA from Fresh Frozen and FFPE Tissues. PLoS ONE.

[11] Lindauer M.L., Wong J., Iwakura Y., Magun B.E. (2009). Pulmonary Inflammation Triggered by Ricin Toxin Requires Macrophages and IL-1 Signaling. J. Immunol..

[12] Breuer K., Foroushani A.K., Laird M.R., Chen C., Sribnaia A., Lo R., Winsor G.L., Hancock R.E.W., Brinkman F.S.L., Lynn D.J. (2013). InnateDB: Systems biology of innate immunity and beyond—Recent updates and continuing curation. Nucleic Acids Res..

[13] Altemeier W.A., Matute-Bello G., Gharib S.A., Glenny R.W., Martin T.R., Liles W.C. (2005). Modulation of lipopolysaccharide-induced gene transcription and promotion of lung injury by mechanical ventilation. J. Immunol..

[14] Chou C.-H., Chang N.-W., Shrestha S., Hsu S.-D., Lin Y.-L., Lee W.-H., Yang C.-D., Hong H.-C., Wei T.-Y., Tu S.-J. (2015). miRTarBase 2016: Updates to the experimentally validated miRNA-target interactions database. Nucleic Acids Res..

[15] Aarøe J., Lindahl T., Dumeaux V., Sæbø S., Tobin D., Hagen N., Skaane P., Lönneborg A., Sharma P., Børresen-Dale A.-L. (2010). Gene expression profiling of peripheral blood cells for early detection of breast cancer. Breast Cancer Res..

[16] Burczynski M.E., Dorner A.J. (2006). Transcriptional profiling of peripheral blood cells in clinical pharmacogenomic studies. Pharmacogenomics.

[17] Lee W., Kim I., Shin S., Park K., Yang K., woo Eun J., Sul H., Jeong S. (2016). Expression profiling of microRNAs in lipopolysaccharide-induced acute lung injury after hypothermia treatment. Mol. Cell Toxicol..

[18] Vaporidi K., Vergadi E., Kaniaris E., Hatziapostolou M., Lagoudaki E., Georgopoulos D., Zapol W.M., Bloch K.D., Iliopoulos D. (2012). Pulmonary microRNA profiling in a mouse model of ventilator-induced lung injury. Am. J. Physiol. Lung Cell Mol. Physiol..

[19] Alipoor S.D., Tabarsi P., Varahram M., Movassaghi M., Dizaji M.K., Folkerts G., Garssen J., Adcock I.M., Mortaz E. (2019). Serum Exosomal miRNAs Are Associated with Active Pulmonary Tuberculosis. Dis. Markers.

[20] Katalan S., Falach R., Rosner A., Goldvaser M., Brosh-Nissimov T., Dvir A., Mizrachi A., Goren O., Cohen B., Gal Y. (2017). A novel swine model of ricin-induced acute respiratory distress syndrome. Dis. Model Mech..

[21] Johnnidis J.B., Harris M.H., Wheeler R.T., Stehling-Sun S., Lam M.H., Kirak O., Brummelkamp T.R., Fleming M.D., Camargo F.D. (2008). Regulation of progenitor cell proliferation and granulocyte function by microRNA-223. Nature.

[22] Pulikkan J.A., Dengler V., Peramangalam P.S., Peer Zada A.A., Müller-Tidow C., Bohlander S.K., Tenen D.G., Behre G. (2010). Cell-cycle regulator E2F1 and microRNA-223 comprise an autoregulatory negative feedback loop in acute myeloid leukemia. Blood.

[23] Mehta A., Baltimore D. (2016). MicroRNAs as regulatory elements in immune system logic. Nat. Rev. Immunol..

[24] Neudecker V., Haneklaus M., Jensen O., Khailova L., Masterson J.C., Tye H., Biette K., Jedlicka P., Brodsky K.S., Gerich M.E. (2017). Myeloid-derived miR-223 regulates intestinal inflammation via repression of the NLRP3 inflammasome. J. Exp. Med..

[25] Neudecker V., Brodsky K.S., Clambey E.T., Schmidt E.P., Packard T.A., Davenport B., Standiford Y.S., Weng T., Fletcher A.A., Barthel L. (2017). Neutrophil transfer of miR-223 to lung epithelial cells dampens acute lung injury in mice. Sci. Transl. Med..

[26] Niu Y., Mo D., Qin L., Wang C., Li A., Zhao X., Wang X., Xiao S., Wang Q., Xie Y. (2011). Lipopolysaccharide-induced miR-1224 negatively regulates tumour necrosis factor-α gene expression by modulating Sp1. Immunology.

[27] Alipoor S.D., Mortaz E., Tabarsi P., Marjani M., Varahram M., Folkerts G., Garssen J., Adcock I.M. (2018). miR-1224 Expression Is Increased in Human Macrophages after Infection with Bacillus Calmette-Guérin (BCG). Iran J. Allergy Asthma Immunol..

[28] Park H.-J., Kim D.-H., Lim S.-H., Kim W.-J., Youn J., Choi Y.-S., Choi J.-M. (2014). Insights into the role of follicular helper T cells in autoimmunity. Immun. Netw..

[29] Jeker L.T., Zhou X., Gershberg K., de Kouchkovsky D., Morar M.M., Stadthagen G., Lund A.H., Bluestone J.A. (2012). MicroRNA 10a marks regulatory T cells. PLoS ONE.

[30] Njock M.-S., Cheng H.S., Dang L.T., Nazari-Jahantigh M., Lau A.C., Boudreau E., Roufaiel M., Cybulsky M.I., Schober A., Fish J.E. (2015). Endothelial cells suppress monocyte activation through secretion of extracellular vesicles containing antiinflammatory microRNAs. Blood.

[31] Hong L., Sharp T., Khorsand B., Fischer C., Eliason S., Salem A., Akkouch A., Brogden K., Amendt B.A. (2016). MicroRNA-200c Represses IL-6, IL-8, and CCL-5 Expression and Enhances Osteogenic Differentiation. PLoS ONE.

[32] Wendlandt E.B., Graff J.W., Gioannini T.L., McCaffrey A.P., Wilson M.E. (2012). The role of microRNAs miR-200b and miR-200c in TLR4 signaling and NF-κB activation. Innate Immun..

[33] Ikari J., Nelson A.J., Obaid J., Giron-Martinez A., Ikari K., Makino F., Iwasawa S., Gunji Y., Farid M., Wang X. (2017). Reduced microRNA-503 expression augments lung fibroblast VEGF production in chronic obstructive pulmonary disease. PLoS ONE.

[34] Park J., Jeong S., Park K., Yang K., Shin S. (2018). Expression profile of microRNAs following bone marrow-derived mesenchymal stem cell treatment in lipopolysaccharide-induced acute lung injury. Exp. Ther. Med..

[35] Ferruelo A., Peñuelas Ó., Lorente J.A. (2018). MicroRNAs as biomarkers of acute lung injury. Ann. Transl. Med..

[36] Korcheva V., Wong J., Corless C., Iordanov M., Magun B. (2005). Administration of ricin induces a severe inflammatory response via nonredundant stimulation of ERK, JNK, and P38 MAPK and provides a mouse model of hemolytic uremic syndrome. Am. J. Pathol..

[37] Beasley M.B. (2010). The pathologist’s approach to acute lung injury. Arch. Pathol. Lab. Med..

[38] Showe M.K., Vachani A., Kossenkov A.V., Yousef M., Nichols C., Nikonova E.V., Chang C., Kucharczuk J., Tran B., Wakeam E. (2009). Gene expression profiles in peripheral blood mononuclear cells can distinguish patients with non-small cell lung cancer from patients with nonmalignant lung disease. Cancer Res..

[39] Goleva E., Jackson L.P., Gleason M., Leung D.Y.M. (2012). Usefulness of PBMCs to predict clinical response to corticosteroids in asthmatic patients. J. Allergy Clin. Immunol..

[40] Severino P., Silva E., Baggio-Zappia G.L., Brunialti M.K.C., Nucci L.A., Rigato O., da Silva I.D.C.G., Machado F.R., Salomao R. (2014). Patterns of gene expression in peripheral blood mononuclear cells and outcomes from patients with sepsis secondary to community acquired pneumonia. PLoS ONE.

[41] Gal Y., Mazor O., Alcalay R., Seliger N., Aftalion M., Sapoznikov A., Falach R., Kronman C., Sabo T. (2014). Antibody/doxycycline combined therapy for pulmonary ricinosis: Attenuation of inflammation improves survival of ricin-intoxicated mice. Toxicol. Rep..

[42] Dobin A., Davis C.A., Schlesinger F., Drenkow J., Zaleski C., Jha S., Batut P., Chaisson M., Gingeras T.R. (2013). STAR: Ultrafast universal RNA-seq aligner. Bioinformatics.

[43] Anders S., Pyl P.T., Huber W. (2015). HTSeq—A Python framework to work with high-throughput sequencing data. Bioinformatics.

[44] Love M.I., Huber W., Anders S. (2014). Moderated estimation of fold change and dispersion for RNA-seq data with DESeq2. Genome Biol..

[45] Pillar N., Bairey O., Goldschmidt N., Fellig Y., Rosenblat Y., Shehtman I., Haguel D., Raanani P., Shomron N., Siegal T. (2017). MicroRNAs as predictors for CNS relapse of systemic diffuse large B-cell lymphoma. Oncotarget.

[46] Yu G., Wang L.-G., Han Y., He Q.-Y. (2012). ClusterProfiler: An R package for comparing biological themes among gene clusters. OMICS.

[47] Ru Y., Kechris K.J., Tabakoff B., Hoffman P., Radcliffe R.A., Bowler R., Mahaffey S., Rossi S., Calin G.A., Bemis L. (2014). The multiMiR R package and database: Integration of microRNA-target interactions along with their disease and drug associations. Nucleic Acids Res..

