# Supplementary material for: Characterization of MicroRNA and Gene Expression Profiles Following Ricin Intoxication
Source: Toxins (Basel). 2019 May 2;11(5):250. doi: 10.3390/toxins11050250 (PMC6563297; doi:10.3390/toxins11050250)
Supplement: Supplementary file 1 [file toxins-11-00250-s001.pdf]

Supplementary Materials: Characterization of MicroRNA and Gene Expression Profiles Following Ricin Intoxication

Nir Pillar, Danielle Haguel, Meitar Grad, Guy Shapira, Liron Yoffe and Noam Shomron

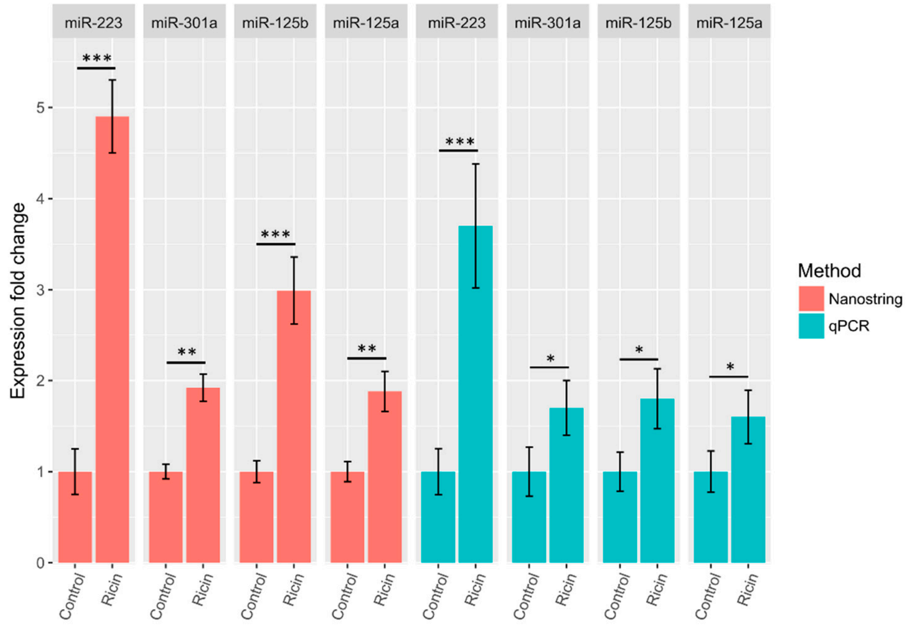

Figure 1. qPCR validation of Nanostring results. Expression of four miRNAs (miR-223, miR-301a, miR-125b and miR-125a), which were differentially expressed in the first Nanostring analysis were tested in qPCR. The qPCR output validated Nanostring results in all miRNAs tested.

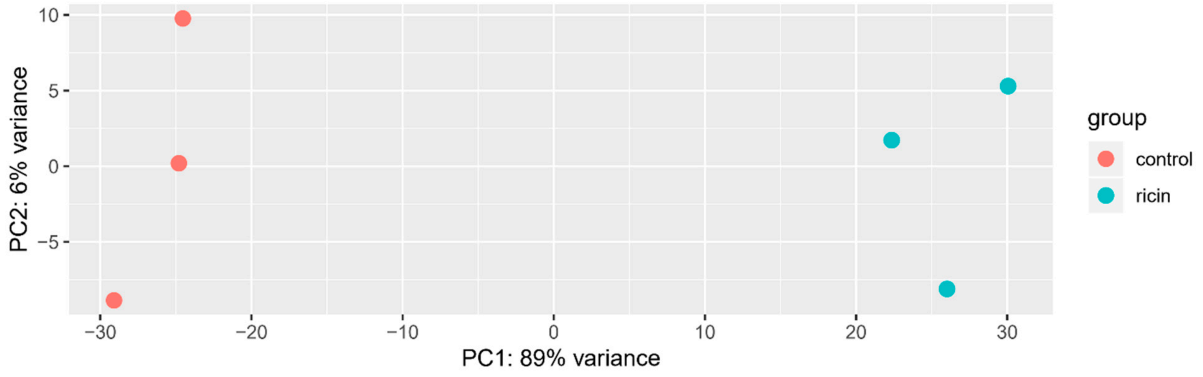

Figure 2. PCA analysis of mRNA expression. The ricin and control groups are clearly distinguished by their mRNA expression profiles, as demonstrated by unsupervised clustering. PCA was performed on the top 500 differentially expressed genes, as advised by Deseq2 [1].

Table S1. (**a**) Expression comparison of all expressed miRNAs between ricin and control groups. (**b**) Expression comparison of all expressed mRNAs between ricin and control groups. Please see the detailed data in excel.

Table S2. Differentially expressed miRNAs between ricin and control groups. Twenty-one differentially expressed miRNAs from the first Nanostring experiment are presented. Five miRNAs which were differentially expressed also in the second Nanostring experiment appear in bold.

miRNAs	Adjusted *p*-value	Mean Control expression	Mean Ricin expression	Target genes with differential expression in RNA seq
**mmu-miR-223**	**1.75×10^{-15}**	**1749.90**	**8623.46**	***Bdp1, Hpcal4, Tspyl3, Dusp8, Lif, Fmnl2, Kcnj3, Zfp467, Dbn1, Ppp4r2, F3, Pcdh17, Pvt1, Ank3, Tppp, Lonrf3, Fam13a, Ptbp2, Ankrd17, Scd1, Pdia6, Mt2, 3110043O21Rik, Rsrc2, Rest, Prkar2b, Serf1, Ntrk2, Jmjd1c, Enc1, Pitpnm3, Sgsm1, Nrep***
mmu-miR-125b	0.00066	3674.64	11015.04	*Ddx3x, Nphp3, Egr2, Slc35d2, Serpine1, Jun, Il1rn, Ajuba*
**mmu-miR-1224**	**0.0000345**	**195.87**	**691.35**	***Rhod, Clk4***
mmu-miR-15b	0.00066	1933.78	3479.09	*Creb5, Itgav, Irgq, Ncl*
mmu-miR-486	0.04087	593.45	1099.08	-
mmu-miR-17	0.01914	1110.21	1676.93	*Cds1, Ranbp2, Map2, Cd28, Zeb2, Celsr2, Nrip1, Nptx1, Cyld, Adcyap1r1, Sqstm1, Piwil2, Cald1, F3, Kif5c, Ugcg, Nr1d2, Shh, Btg2, Fam227a,Pum1, Pvr, Mctp2, Slc17a7, Ankrd17, Pim3, Elovl6, Srcin1, Dpysl2, Celf2, Timp1, Rsrc2, Kbtbd8, Ryr2, Zfp217, Dio2, Slc7a2, Slc1a4,Pappa, Klf10, Ajuba, Ppp2r2c, Pcf11, Skil, Ptpn11, Cbx2*
mmu-miR-125a	0.00025	1611.51	3032.46	*Ptpn18, Il1rn*
mmu-miR-342	0.00135	1418.91	1028.98	*Prrg4, Dapk1, Creb5, Wdr43, Fubp1, Ociad2, Dzank1, Vav1, Ppp1r26, Arhgap23, Pttg1, Clec7a, Itgam*
mmu-miR-301a	0.00007	509.94	981.35	*Nkrf, Rhod, Irf1*
mmu-miR-150	0.000259	5345.92	4299.96	*Zfp488, Cbl, Dixdc1, Egr2, Pik3c2a, Depdc5, Zfp36l1, Zfp568, Ybey, Ripk2, Myb, Arhgap23*
mmu-let-7b	0.00635	24162.91	19612.64	-
**mmu-miR-10a**	**0.00139**	**1514.00**	**1318.02**	***Ptpn2, Ccl9, Cenpl, Car8, Mmp25, Sgms2, Eno2, Rnd1, Tnfrsf10b, Fam227a, Tbc1d24, Gaa, Aco1, Mcc, Stam, Decr2, Rhbdl3, Ajuba, Shroom1, Ramp1, Klhl41, Ecm2***
**mmu-miR-503**	**0.02971**	**190.89**	**23.16**	***Creb5, Ncl, Inhbb***
mmu-miR-24	0.04087	358.07	550.79	*Lrtm2, Slc15a4, Mdk, Kcnj3, L1cam, Lmtk2, Pianp, Ncl, Birc5, Plch1, Nfat5, Trib3, Cyld, Adcyap1r1, Atf3, AU019823, Vav1, Rab3d, Eno2, Mak16, Ugcg, Il6st, Skida1, Slc25a25, Ank3, Tppp, Slc35d2, Slc4a8, Snhg11, Clmn, Zfp697, Gap43, Smad3, Klf6, Gnal, Zmym3, Atp1a2, Cnbd2, Sptbn2, Tspyl4, Gpt2*
mmu-miR-1944	0.000258783	6935.98	6191.66	-
mmu-miR-22	0.011718985	25361.37	23308.62	*Irf8, Tnfrsf10b*
mmu-miR-200a	0.016465377	2016.24	1883.19	*Qk, Fat3, Tgfb2, Zeb2, Fam227a, Ppp4r2*

mmu-let-7i	0.029713352	24251.19	23562.42	*Slc2a6, Chd7, Zfp462, Dusp1, Slc25a23, Cat, Fnip1, Zeb2, Sema4f, Bsg, Cpeb2, Atp2b2, Cald1, Tnfrsf26, Nol8, Ago3, Tbc1d24, Frmd5, Slc4a8, Slco5a1, Srcin1, Epha4, Syt5, Mex3a, Rffl, Gnal, Thbs1, Kifc2, Slc4a7, Sptbn2, Skil, Patl1, Tspan2*
**mmu-miR-200c**	**0.002702735**	**2223.93**	**1981.17**	***Jun, Ikzf5, Map2, Sox2, Zeb2, Mgat3***
mmu-miR-200b	0.026253042	4987.96	4763.18	*Jun, Cited2, Ikzf5, Map2, Zeb2, Mgat3*
mmu-miR-21	0.035582713	5435.07	5400.57	-

References

1. Love, M.I.; Huber, W.; Anders, S. Moderated estimation of fold change and dispersion for RNA-seq data with DESeq2. *Genome Biol.* **2014**, *15*, 550.
